# Editorial: Mitochondria and Endoplasmic Reticulum Dysfunction in Parkinson’s Disease

**DOI:** 10.3389/fnins.2019.01171

**Published:** 2019-11-08

**Authors:** Sandeep Kumar Barodia, Krishnan Prabhakaran, Smitha Karunakaran, Vikas Mishra, Victor Tapias

**Affiliations:** ^1^Center for Neurodegeneration and Experimental Therapeutics, Birmingham, AL, United States; ^2^Department of Biology, Norfolk State University, Norfolk, VA, United States; ^3^Centre for Brain Research, Indian Institute of Science, Bangalore, India; ^4^Department of Pharmaceutical Sciences, Basanaheb Bhirao Ambedkar University, Lucknow, India; ^5^Feil Family Brain and Mind Research Institute, Weill Cornell Medicine, New York, NY, United States

**Keywords:** Parkinson's disease, mitochondria, endoplasmic reticulum, α-synuclein, PINK1, parkin, oxidative stress, MAMs

Endoplasmic reticulum (ER) and mitochondria are distributed in close communication via a dynamic ER-calcium (Ca^2+^) mitochondria interconnection and regulate a plethora of vital cellular functions, including Ca^2+^ homeostasis, mitochondrial transport and dynamics, bioenergetics, ER stress, apoptotic signaling, and inflammation (Erpapazoglou et al., 2017). Alteration in the ER-mitochondria communication adversely affects overall physiology of the cell (Gómez-Suaga et al., 2018). ER-mitochondria communication is also involved in lipid transport, suggesting that lipidomic approach may be useful to study the potential mechanisms leading to impaired neuropeptidergic signaling (Valadas et al., 2018). Mitochondria-associated membranes (MAMs) are defined as specialized subdomains connecting ER and mitochondria in order to regulate physiological functions, maintain Ca^2+^ signaling and other vital cellular processes (Rodríguez-Arribas et al., 2017). Neurons are highly dependent on MAMs to exchange metabolites and signaling molecules between ER and mitochondria, suggesting that altered function of MAMs due to toxin insults such as rotenone and manganese could play a crucial role in the pathogenesis of neurodegenerative diseases, including Parkinson's disease (PD) (Krols et al., 2016; Harischandra et al.; Ramalingam et al.; Valdinocci et al.). Modifications in the communication between ER and mitochondria cause a reduction in mitochondrial Ca^2+^ homeostasis in several animal models of neurodegeneration, such as PD, an age-dependent neurodegenerative disorder characterized by the progressive loss of dopamine (DA)-producing neurons in the substantia nigra (Paillusson et al., 2016; Lee et al., 2018). Several cellular mechanisms have been identified to be involved in the DAergic neuronal death, including mitochondrial dysfunction, impaired bioenergetics, oxidative stress, autophagy and impaired intracellular Ca^2+^ homeostasis in patient-derived cell models of PD (González-Casacuberta et al.; Segura-Aguilar). However, mechanisms underlying how organelle crosstalk (especially between mitochondria and ER) could affect the progression of pathogenesis in PD still remain unknown. ER stress activates unfolded protein response through the upregulation of the ER chaperone GRP78 and caspases as well as evokes Ca^2+^ flux that induces mitochondrial dysfunction and associated loss of DA neurons (Arduíno et al., 2009; Baek et al.). Interestingly, increased ROS production through PERK/eIF2α/ATF4/CHOP pathway of UPR and concomitant alteration of the mitochondrial network morphology have been reported in PARK20 fibroblasts (Amodio et al.). Emerging evidence supporting significance of altered ER–mitochondria communication suggests that damaged ER–mitochondria signaling could be a potential therapeutic strategy to treat neurodegenerative diseases.

The present Research Topic is an effort to showcase the significance of MAM in PD pathogenesis. Here, we discuss the recent findings in PD research with main focus on molecular and cellular mechanisms involving mitochondria and ER. Pathophysiological significance of ER-mitochondria interaction has been demonstrated in the case of PD-related genes, such as α-synuclein (α-syn) (Guardia-Laguarta et al., 2014), DJ-1 (Ottolini et al., 2013), PINK1 (Celardo et al., 2016; Gelmetti et al., 2017), and Parkin (Van Laar et al., 2015; Celardo et al., 2016; Gautier et al., 2016; Gelmetti et al., 2017; Zheng et al., 2017). Several clinical cases with diagnosed PD show a well-defined Lewy body pathology (Cookson et al., 2008), which are composed of α-syn. Protein aggregation and imbalanced cellular proteostasis are key factors leading to accumulation of misfolded α-syn (Lehtonen et al.). Within neurons, α-syn is diversely localized to cytosolic and membrane compartments including synaptic vesicles, mitochondria and the ER (Guardia-Laguarta et al., 2015; Colla). Membrane localization of α-syn is well-targeted to lipid rafts (detergent-resistant membranes) that are enriched in cholesterol and acidic phospholipids (Fortin et al., 2004). Interestingly, a subpopulation of α-syn is shown to be enriched in MAM fraction in immortalized cell lines and in the mouse and human brain (Poston et al., 2013; Guardia-Laguarta et al., 2014; Paillusson et al., 2016). Certainly, identification of the A53T mutation in the gene encoding for α-syn (*SNCA*) provides us better understanding of both the genetics and the neuropathology of PD (Polymeropoulos et al., 1997). It has been demonstrated that A53T mutant showed a decreased association with MAM and an elevated mitochondrial fragmentation, as compared to wild-type α-syn (Guardia-Laguarta et al., 2014). Moreover, overexpression of either wild-type or mutant α-syn decreases ER–mitochondria contacts (Paillusson et al., 2016). Thus, substantial accumulation of α-syn aggregates could be linked to the loss of function of this protein at the MAMs. Interestingly, subcellular localization of α-syn to MAM could be related to both normal and pathological states (Guardia-Laguarta et al., 2014, 2015). A recent study demonstrated that α-syn binds to VAPB (an ER-mitochondria tethering protein) to disrupt Ca^2+^ homeostasis and mitochondrial ATP production (Paillusson et al., 2016).

PINK1/Parkin-mediated mitophagy could be an underlying mechanism of nigral DA neuron death in PD (Thomas et al., 2011; Kane et al., 2014; Barodia et al., 2017). ER-mitochondria contact sites were shown to constitute the initiation sites for this process (Yang and Yang, 2013). During mitophagy, PINK1 and BECN1 re-localize at MAM, which induces ER-mitochondria tethering and autophagosome formation (Gelmetti et al., 2017). Parkin expression was significantly increased in the MAM fraction of neurons following glutamate excitotoxicity (Van Laar et al., 2015), which also ubiquitylated several proteins of the ER-mitochondria interface including Mfn2, VDACs and Miro (Sarraf et al., 2013; Pickrell and Youle, 2015). Parkin may regulate ER-mitochondria communication via Mfn2 (Basso et al., 2018). Mitochondrial and ER stress results in an upregulation of Parkin levels via ATF4 (Bouman et al., 2011). ER-mitochondria communication was reported to be increased in fibroblasts from patients with *PARK2* or *PARK6* mutations compared to control group (Celardo et al., 2016; Gautier et al., 2016). This alteration was associated with higher mitochondrial Ca^2+^ absorption, upon IP_3_R stimulation. Similar structural changes were observed in MEFs from *PARK2* knock-out mice (Gautier et al., 2016). Parkin has recently been reported to co-regulate ER-mitochondria communication together with the transcription factor peroxisome proliferator activated receptor g coactivator 1a (PGC-1α), a key modulator of mitochondrial biogenesis (Zheng et al., 2017). ER–mitochondria associations have also been linked to the formation of the inflammasome. Cellular stress in neurodegenerative diseases are detected by the innate immune system through pattern recognition receptors (Paillusson et al., 2016). Reactive oxygen species (ROS) from mitochondria are one signal for activation of the NLRP3 inflammasome (Abais et al., 2015). Elevated ROS generation led to NLRP relocation to MAM, which may provide a mechanism whereby NLRP senses damage mitochondria to activate the inflammasome (Zhou et al., 2011). Due to the importance of MAMs in understanding the fundamental mechanisms of PD pathogenesis and their potential use as a therapeutic approach, further research is needed to investigate on the communications between the ER and mitochondria.

## Author Contributions

SB collected the relevant references and wrote the manuscript. VT, KP, SK, and VM edited the manuscript and provided thorough reviews on the manuscript.

### Conflict of Interest

The authors declare that the research was conducted in the absence of any commercial or financial relationships that could be construed as a potential conflict of interest.
